# 

**Published:** 1913-12-20

**Authors:** 


### Answers to Correspondents.

The Secretary of a local medical benefit club asks for information as to the working expenses of similar clubs. There is no ready access to the accounts of such clubs, but we understand that the working expenses are generally very small indeed, *i.e.*, from 5 to 10 per cent., the secretary often acting in an honorary capacity.

### A HOSPITAL'S SERVICES REWARDED.

It is noted with pleasure that the Cheshire Lines Committee has recognised the services of the medical officers who gave their assistance at the scene of the recent disaster at St. James's Station, Liverpool, and that a sum of two hundred guineas has been awarded to the Royal Southern Hospital as a token of the Committee's appreciation of the valuable services rendered by that institution.

### Appointments.

Mr. E. M. Mahon, M.B., B.S., has been appointed house surgeon to the Female Lock Hospital, Harrow Road, W.

Mr. P. C. P. Ingram, M.B., B.S. Lond., has been appointed hon. anæsthetist, Royal Government Hospital, Newport.

Dr. R. D. Muir has been appointed one of the London County Council's district medical officers in succession to Dr. W. Willes, resigned.

Mr. H. K. V. Soltau, M.B., L.R.C.P. Lond., M.R.C.S. Eng., has been appointed house surgeon at the Birmingham and Midland Hospital for Women.

Mr. Lancelot Bromley, M.A., M.B., M.C. Cantab., F.R.C.S. Eng., has been appointed surgeon to outpatients, Victoria Hospital for Children.

Dr. Arthur Riley, of the Brompton Hospital Sanatorium at Frimley, Surrey, has been recommended for the post of tuberculosis dispensary officer for Bury, Lancashire.

Mr. Alex. G. McLean, M.B., Ch.B., of Aberdeen University, has been appointed house physician to the West End Hospital, Welbeck Street, and Mr. Hildred B. Carlill, M.D., M.R.C.P., has been re-appointed clinical assistant to Dr. Purves Stewart.

### Gifts and Bequests.

The Mercers' Company have made a grant of £20 to the National Association for the Feeble-Minded.

The Royal Dental Hospital, Leicester Square, has received £5 as a Christmas donation from the Queen.

A bequest of £20 to the funds of the Leicester Royal Infirmary is contained in the will of the late Miss M. S. Palmer, of Burrough.

The Government of the Gold Coast has voted £250 to the fund raised by Mr. Austen Chamberlain for the extension and development of the London School of Tropical Medicine.

Mr. John Anderson, of Porchester Terrace, W., late of 33 Bryanston Square, W., and formerly of Hamburg and of Leith, has bequeathed £2,000 each to the Middlesex Hospital and the New Hospital for Women.

The Right Hon. G. W. Palmer, who left estate of the value of £765,676, has bequeathed to the Royal Berkshire Hospital £5,000, and £500 each to the Reading Dispensary and the Children's Cottage Hospital, Cold Ash, near Newbury.

Among the latest contributions received at the Bank of England for King Edward's Hospital Fund for London are the following annual subscriptions : Sir Savile Crossley, Bart., £250 (to capital); Army and Navy Co-operative Society, Ltd., £52 10s.; Sir John Craggs, £25; Mr. Frederick M. Fry, £25.

Mrs. Sarah Holmes, of Sussex Place, Hyde Park, widow of Mr. Timothy Holmes, left estate of which £23,144 is net personalty. Under the will of her late husband, as she survived him, she inherited all his estate, but had she predeceased him he had left numerous legacies, including £1,000 to St. George's Hospital and £100 to the Invalid Children's Aid Association, which she now directed should be paid.

**"THE HOSPITAL" Building, 28 & 29 Southampton St., Strand, London, W.C.**

